# Impact of glycemic variability on cognitive impairment, disordered eating behaviors and self-management skills in patients with type 1 diabetes: study protocol for a cross-sectional online study, the Sugar Swing study

**DOI:** 10.1186/s12902-022-01191-4

**Published:** 2022-11-18

**Authors:** Sylvain Iceta, Léonie Sohier, Catherine Bégin, Anne-Sophie Brazeau, Rémi Rabasa-Lhoret, Claudia Gagnon

**Affiliations:** 1 Research Center of the Quebec Heart and Lung Institute, Québec, QC Canada; 2 Department of Psychiatry and Neurosciences, Laval University, Québec, QC Canada; 3 School of Psychology, Laval University, Québec, QC Canada; 4 Centre d’expertise, Poids, Image Et Alimentation (CEPIA), Québec, QC Canada; 5 School of Human Nutrition, McGill University, Montreal, QC Canada; 6 Montreal Institute for Clinical Research, Montreal, QC Canada; 7 Department of Nutrition, Montreal University, Montreal, QC Canada; 8 CHU de Québec-Université Laval Research Centre, Québec, QC Canada; 9 Department of Medicine, Laval University, Québec, QC Canada

**Keywords:** Type 1 Diabetes, Cognition, Eating Behaviors, Disordered Eating, Attention, Impulsivity, Continuous glucose monitoring, Glycemic Variability, Self-management

## Abstract

**Background:**

People living with type 1 diabetes (PWT1D) are at increased risk for impairments in brain function, which may impact on daily life. Cognitive impairments in PWT1D might contribute to increasing eating disorders, reducing self-management skills, and deteriorating glycemic control*.* Glycemic variability may be a key determinant of disordered eating behaviors, as well as of cognitive impairments. The main objective of this study is to better understand the impact of glycemic variability in disordered eating behaviors and cognitive impairment, and its consequences on self-management skills in PWT1D.

**Method:**

We aim to recruit 150 PWT1D with 50% of men and women in this cross-sectional study. Participants will record their glycemic variability over a 10-day period using a continuous glucose monitoring system (CGMS) and track their dietary intakes using image-assisted food tracking mobile application (2 days). Over four online visits, eating behaviors, diabetes self-management’s skills, anxiety disorders, depression disorder, diabetes literacy and numeracy skills, cognitive flexibility, attention deficit, level of interoception, and impulsivity behaviors will be assessed using self-reported questionnaires. Cognitive functions (i.e., attention, executive functions, impulsivity, inhibition and temporal discounting), will be measured. Finally, medical, biological and sociodemographic data will be collected. To further our understanding of the PWT1D experience and factors impacting glycemic self-management, 50 PWT1D will also participate in the qualitative phase of the protocol which consist of individual in-depth face-to-face (virtual) interviews, led by a trained investigator using a semi-structured interview.

**Discussion:**

This study will contribute to highlighting the consequences of blood sugar fluctuations (i.e., "sugar swings"), in daily life, especially how they disrupt eating behaviors and brain functioning. A better understanding of the mechanisms involved could eventually allow for early detection and management of these problems. Our study will also seek to understand the patients' point of view, which will allow the design of appropriate and meaningful recommendations.

**Trial registration:**

ClinicalTrials.gov, NCT05487534. Registered 4 August 2022.

## Background

The discovery of insulin 100 years ago deeply transformed the face of type 1 diabetes (T1D) from a rapid life-threatening disease to a chronic condition [1]. By becoming a long-term disease, T1D brings new challenges to physicians including the management of chronic complications. Prevention screening and management of classical micro- and macrovascular complications of T1D (e.g., retinopathy, nephropathy, neuropathy and cardiovascular complications) have been the focus of much research, establishing the crucial importance of the glucose control for primary and secondary prevention [2]. However, other complications of T1D such as brain impairment, that may significantly affect quality of life, are still understudied and thus, under-recognized and poorly addressed.

It has been established that T1D in children affects brain development and that impairment in brain structure and function may increase over time [3]. In elderly patients with T1D (PWT1D), almost 50% experience significant cognitive impairment [4]. In a recent study, Jacobson et al. found that adults with a long history of T1D have reduced brain gray and white matter volume [5]. They observed that the brain volumes of patients with T1D (PWT1D) appeared like those of individuals without diabetes who are 4–9 years older, and thus in relatively young elderly patients (age median: 60 yr) [5]. In elderly PWT1D, almost 50% experience significant cognitive impairment [4]. Although of excellent quality, the tests used in most studies detect cognitive problems that are already clinically perceptible [6, 7]. Less is known about “subclinical” or more specific brain function impairment, and their functional relevance. Such “subclinical” impairment may affect a higher number of PWT1D, and their impact on daily life is likely underestimated. Studies suggest that PW1TD have a specific pattern of cognitive impairment: 1) that predominantly affects global intelligence, attention, psychomotor speed, executive function and cognitive flexibility [8], 2) among executive functions, inhibition, working memory and set-shifting (i.e., the ability to alternate between one task and another) might be particularly affected [9], 3) cognitive impairment patterns and their consequences may differ by sex [10]. However, observed effect sizes were weak to mild and the literature remains scarce and inconsistent.

Diabetes self-management aiming for strict glycemic targets requires insulin therapy (e.g., multiple daily injections or insulin pump therapy) and active participation from PWT1D, and sometimes of their relatives. It has been estimated that life with T1D requires up to 180 health-related decisions per day. The most used factor to assess the severity of T1D is the HbA1c. While HbA1c accurately reflects long-term glycemic control, it only partially reflects the day-to-day variations that underlie T1DM management and dietary choices. Glycemic variability has been well studied and has emerged as a more meaningful measure of glycemic control. Based on continuous glucose monitoring, glycemic variability measurements reflect either short-term (with-day and between-day variability) or long-term blood glucose levels fluctuations (i.e., sugar swing) [11]. Higher glycemic variability has been associated with an increased risk of macro- and microvascular complications, as well as a higher mortality rate [11]. However, studies on cognitive impairments or disordered eating in patients with T1DM rarely use continuous glucose monitoring system (CGMS) and glycemic variability measurement (e.g., coefficient of variation).

Insulin restriction or omission to control weight is a well-known and frequent phenomenon, affecting up to 40% of PWT1D [12, 13]. Such behavior can culminate in a particular form of disease close to anorexia nervosa called diabulimia [13]. This disorder results in dramatic increases in hemoglobin A1c (HbA1c) levels [13]. Purging or binge eating behaviors are also frequent disordered eating behaviors (DEB) in PWT1D (associated or not with restrictive eating behaviors) [14]. Moreover, the presence of binge eating behavior seems to be associated with higher anxiety and depression levels [15]. It has been demonstrated, and even mathematically modeled, that appetite is partly controlled by glycemic levels (e.g., hypoglycemia as a hunger trigger and hyperglycemia as a hunger suppressor) [16]. However, these findings are mainly based on animal studies and the impact of glycemic variability on eating behavior, and vice versa, needs to be further studied in PWT1D. Recently, Zhou et al. found that total daily energy intake does not seem to impact the glycemic variability index in PWT1D [17]. As glycemic variability is a dynamic phenomenon over the day and between days, its impact on food behavior is not likely to be detected by a change in the total amount of food consumed. More accurate reporting of both eating behavior (including loss of control eating and binge eating behaviors) and macronutrient intake (especially carbohydrate intake) may be more relevant to evaluate the consequences of glycemic variability. Moreover, we did show that inaccurate carbohydrate counting is frequent and associated with higher daily blood glucose variability in PWT1D [18]. Thus, diabetes-related numeracy will be considered a potential confounder in our study. Treasure et al. made the assumption that DEB may result in larger glycemic fluctuations [19]. Higher glycemic fluctuations may in turn affect cognitive function, potentially leading to more difficulties to maintain adequate blood glucose levels and eating behavior, thus setting up a vicious circle. Indeed, cognitive functions are highly involved in eating behavior, as they allow the modulation of food intake [20]. We recently published a meta-analysis in which we established that binge eating disorder is significantly associated with impairments in cognitive flexibility, inhibitory control, attention, and planning [21]. Thus, some of the cognitive impairments identified in PWT1D are the same as those identified in binge eating disorder. This overlap may account for DEB occurrence in PWT1D. However, there is currently limited or no evidence of the association between cognitive impairment, DEB and glycemic variability in PWT1D. The relationship between glycemic variability and DEB is challenging to establish since it could be mediated by impairment in executive functions (especially inhibitory control) and/or in interoceptive awareness (i.e., the ability to perceive body signals). Considering such parameters as potential mediators of this relationship might provide the opportunity to address this challenge.

Current evidence also suggests that cognitive impairment may affect treatment adherence and diabetes self-management in PWT1D [22, 23], with the potential to worsen glycemic levels. Poorly controlled diabetes may in turn worsen further cognitive functions [23, 24]. Using questionnaires, but not cognitive tasks, Vloemans et al. found that poorer executive functions are associated with higher HbA1c over time in youth with T1D [25]. The underlying mechanisms responsible for cognitive impairment in PWT1D are difficult to characterize, partly due to numerous confounding factors (e.g., age, HbA1c level, psychiatric comorbidities). Chronic hyperglycemia, longer diabetes duration, and the presence of micro- and macrovascular complications have all been associated with a higher risk of cognitive impairment in PWT1D [3]. Severe and/or repeated hypoglycemia episodes might also be involved in the increased risk of dementia in PWT1D [26]. While most studies focused on acute hypoglycemia, few have assessed glycemic variability, which better considers the broad spectrum of hyper/hypoglycemia frequency, intensity and duration. Another key limitation in current literature is the lack of reporting either hypoglycemia or hyperglycemia episodes during testing, even though it can have a direct effect on performance [24]. These limitations will be addressed in the present study. Based on the SEARCH cohort, Nip et al. were the first to explore the association between insulin sensitivity (insulin sensitivity score estimated from clinical measures including waist circumference, HbA1c, and triglyceride levels) and DEB. In PWT1D, they found that the presence of DEB was associated with greater insulin resistance and a higher body mass index (BMI) [27]. In patients with obesity, we have shown that higher dysfunctional adipose tissue is associated with greater insulin resistance and a higher severity of binge eating behaviors [28]. Hence, accounting for insulin resistance as a potential mediator of the association between glycemic variability and DEB (e.g., binge eating behavior) or cognitive dysfunction could unravel the importance of insulin resistance, even in T1D. This is especially critical since “Double Diabetes” (i.e., PWT1D displaying insulin resistance) prevalence is estimated at 30–50% of PWT1D and keeps on rising [29–31]. These data highlight the significance of this trend and the need to take into account insulin resistance as a potential mediator.

### Study Aim

We aim to better understand the impact of glycemic variability in DEB and cognitive impairment, and its consequences on self-management skills in PWT1D. The primary objective of this study (the Sugar Swing Study) is to compare cognitive function, DEB, and self-management skills in PWT1D with a high glucose variability (i.e., a coefficient of variation [CV] > 36% over a 10-day of CGMS) versus those with a low glucose variability (i.e., CV < 36% [32]). Secondary objectives are: 1) to explore the associations between cognitive impairment, DEB, and self-management skills with other CGMS parameters (glucose variability as continuous variable, % glucose time in range, time below or above range, mean amplitude of glycemic excursions, standard deviation), adiposity (BMI, waist circumference), and insulin resistance markers (estimated Glucose Disposal Rate, eGDR); 2) to determine the potential role of cognitive dysfunction, interoception awareness level and insulin resistance in the relationship between glucose variability and DEB; and 3) to further our understanding of the PWT1D experience and factors impacting glycemic self-management, throughout patient’s perspectives, using a qualitative design.

We hypothesize that, among PWT1D: 1) higher glycemic variability is associated with higher DEB and poorer cognitive function, and that differences exist between sexes; 2) higher DEB and poorer cognitive function are associated with lower self-management skills; and 3) cognitive impairment, interoception awareness and insulin resistance may mediate the relationship between glycemic variability and DEB.

## Methods/design

### Design and participants

**Fig. 1:**
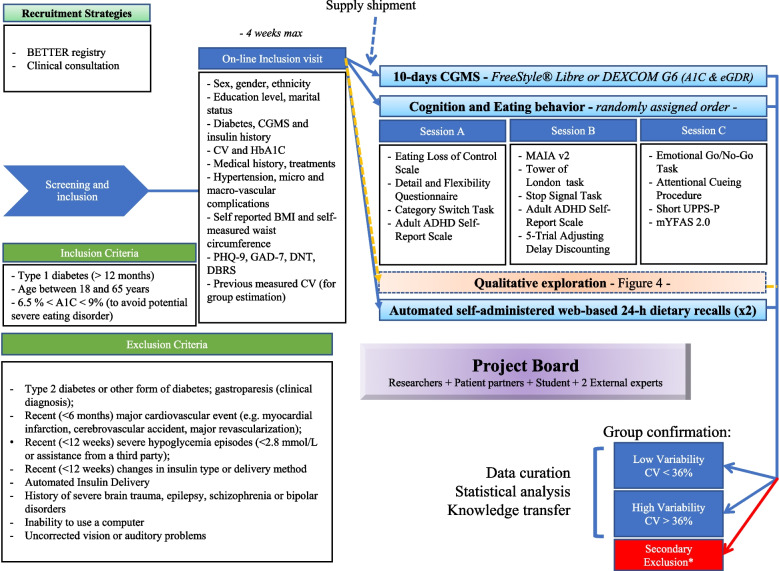
Schematic representation of the methodology for the “Sugar Swing study”. *Legend:* CGMS: continuous glucose monitoring system; CV: coefficient of variation; BMI: Body Mass Index; PHQ-9: Patient Health Questionnaire; GAD-7: General Anxiety Disorder; DNT: Diabetes Numeracy Test; DBRS: Diabetes Behavior Rating Scale; T1-DDS: Type 1 Diabetes Distress Scale; EDE-Q: Eating Disorder Examination Questionnaire MAIA v2: Multidimensional Assessment of Interoceptive Awareness; mYFAS: modified Yale Food Addiction Scale; ADHD: Attention Deficit Hyperactive Disorder; eGDR: estimated Glucose Disposal Rate; *e.g., CGMS < 10 days, missing data > 15%

Study design is detailed in the appendix (Fig. 1 for design and inclusion/exclusion criteria). We will aim to recruit 150 PWT1D with 50% of men and women, and we will recruit participants, in part, from visible minority populations. Participants will be recruited through endocrinology clinics in Québec City and Montreal, Canada and through the BETTER registry [33]. All assessments (inclusion visit, questionnaires, or tasks) will be completed online through secured platforms.

### Quantitative measures

#### Inclusion visit

During this visit conducted online (e.g., using Zoom® or Teams®), the following data will be collected: socioeconomic data, ethnicity, education level, marital status, self-identified gender, diabetes duration, age of diabetes onset, modality of insulin delivery (pump vs injections; closed loop delivery systems are excluded from the present study) and total daily insulin dose, modality of glucose-monitoring (capillary glucose testing or CGMS), duration of CGMS usage, number of severe hypoglycemic episodes over the last year and diabetic ketoacidosis, current medications (included use of psychotropic drugs to be considered as a potential confounding factor), number and type of chronic diabetes micro and macrovascular complications, and medical comorbidities (including hypertension); measured CV based on previous CGMS (e.g., data uploaded during the visit) and last available HbA1c (max 6 months); weight, height and waist circumference (self-measured by the participant as previously described [33]). During this visit, self-management’s skills will be assessed using the *Diabetes Behavior Rating Scale* and the *Type 1 Distress Scale* (a 28-item self-administered questionnaire [34]). Using short screening tests, anxiety (*GAD-7*, 7 items), depression (*PHQ-9*, 9 items) and diabetes numeracy (*Diabetes Numeracy Test*, 15 items, used as proxy for literacy) will be assessed.

#### Glucose variability and insulin resistance

CGMS will be performed over a 10-day period using a Dexcom G6 and data reported following the international consensus [35, 36]. Required material and assistance will be provided by mail if needed. In the province of Quebec (Canada) both the Freestyle Libre and Dexcom are eligible for reimbursement by the public insurance plan for most PWT1D. For the few patients who would not have access to CGMS yet, we will provide the equipment for the duration of the study and a set of sensors will be sent to all participants, as a courtesy for participating in the study. Glucose Management Indicator (GMI) will be used to estimate A1C from CGMS during the study time. The estimated Glucose Disposal Rate (eGDR) will be computed (eGDR is a validated tool for estimating insulin sensitivity in T1D; eGDR = 21.158 + -0.09 × waist circumference − 3.407 × hypertension [defined as 0 = no, 1 = yes] − 0.551 × HbA1c). During the period of CGMS recording, PWT1D will be invited to track their dietary intakes using image-assisted food tracking mobile application over 2 days at random (one weekday and one weekend day) with the Keenoa® application [37].

#### Eating behavior and cognitive tasks

Self-report questionnaire and cognitive tasks will be distributed over 3 sessions of 30 to 45 min each. The session will be scheduled during the 10-day of CGMS measure. Session order will be randomly determined for each participant and each session will start with a short video describing the objectives and procedures. Each session will cover more than one psychological or cognitive domain in order to get different timepoint assessments for each construct.

Glycemic value before and after each session will be collected from CGMS (if participants are experiencing or have experienced hypoglycemia within the last hour, they will be asked to postpone the session) and participants will be asked to perform each session in the same condition (i.e., time of day, location, computers). ***Session A:*** Eating behavior will be assessed by the *Eating Disorder Examination Questionnaire* (a 36-item questionnaire to measure of the range and the severity of eating disorder feature over the past four weeks [38]) and by the *Binge Eating Scale* (a 16-item questionnaire assessing the severity of binge eating behavior [39]). During this session, cognitive flexibility will be assessed by the *Detail and Flexibility Questionnaire* (a 24-item questionnaire validated in eating disorder and exploring difficulties in flexibility/cognitive rigidity and attention to detail/weak central coherence [40]). Attention will be measured by the *Category Switch Task* (a test of divided attention with two simple categorization tasks [41]) and attention Deficit Hyperactive Disorder will be screened by the *Adult ADHD Self-Report Scale* (6 items) [42]. ***Session B:*** The level of interoception will be assessed by the *Multidimensional Assessment of Interoceptive Awareness version 2* (a 32-item questionnaire to measure the conscious level of interoception’s multiple dimensions [43]). This session will also include 3 neurocognitive tasks: the *Tower of London task* (an assessment of executive functioning with a focus on planning abilities [44]), the *Stop Signal Task* (an assessment of impulsivity through the time needed to inhibit a prepotent response [45]), and the *5-Trial Adjusting Delay Discounting* (a quick procedure to estimate delay discount rates of monetary rewards [46]). ***Session C:*** During this session, we will conduct an *Emotional Go/No-Go Task* (a measure of inhibition leverages by emotion recognition and regulation [47]) and the *Attentional Cueing Procedure* (a conditional learning paradigm to study the effects of threat stimuli on capturing and holding attention). Behavioral impulsivity will be measured by the *Short UPPS-P Impulsive Behavior Scale* (a 20-item self-report questionnaire assessing impulsivity as a multifaceted construct [48]). Susceptibility to food reward will be estimated using the *modified Yale Food Addiction Scale 2.0* (a 13-item questionnaire assessing food addiction [49]).

#### Qualitative measures

To further our understanding of the PWT1D experience and factors impacting glycemic self-management, individual in-depth face-to-face (virtual) interviews will be led by a trained investigator (research coordinator) using a semi-structured, open-ended guide. The semi-structured interview protocol will be designed with the involvement of patients’ partners, psychologists and diabetologists. Questions about facilitators and barriers associated with glycemic management, with particular attention to those associated with cognitive function and eating behaviors, will be asked. Participants will also be questioned about the type of therapeutic approaches they need to help them cope with T1D. Interviews will be recorded, transcribed verbatim, and coded. Inductive thematic content analysis will be conducted independently by 2 members of our team (including the research coordinator with expertise in qualitative analysis) using NVivo qualitative research software. Based on the criteria of Malterud et al. [50] and previous experiences of our group, a provisional number of 50 participants (i.e., 25 PWT1D with high glucose variability and 25 PWT1D with low glucose variability) is considered a conservative initial assessment. Analyses regarding data completeness will be repeated every 3 interviews until no new relevant themes emerge (i.e., until saturation). The quantitative and qualitative data will then be juxtaposed, to deepen our understanding of appropriate and meaningful interventions and recommendations for individuals with T1D.

#### Recruitment procedures

Participants will be recruited through the endocrinology clinics in Québec City and Montreal, Canada and through the BETTER registry [33]. The multidisciplinary of the researchers and the participation of patients’ partners will contribute to the feasibility. The involvement of specialists in psychology, neuroscience, eating behaviors and nutrition, as well as of endocrinologist experts in T1D makes this project unique and ensures that all the fields covered by this study are accurately assessed. The synergy of the research professionals involved in the study will strengthen the process.

#### Feasibility

As illustrated in a recent publication by Pyatak et al. [51], remote data collection for psychological testing and CGMS can be successfully implemented by mailing study material, conducting study visits via videoconferencing, and using progressive financial compensation. At the inclusion visit, CGMS records and dietary tracking will be compensated by a $30 stipend, each session by a supplemental $15 and at the end of the procedure, a supplemental $10 stipend will be considered for full completion (i.e. < 10% of missing data). These may seem rather high, but the psychological sampling in this study is demanding and therefore such amount is more likely to ensure the feasibility of the procedure. However, it is important to also provide for the possibility of conducting the evaluation in presential session to ensure that people who do not have access to sufficient technology are not excluded from the study, therefore participants will have the option of completing the sessions at the hospital.

#### Data management

Data will be recorded and stored electronically in REDCap, hosted at the Centre Hospitalier Universitaire de Québec – Laval University. Data will be routinely checked for missing and/or erroneous values. A quality check of recorded data will be provided before the end of participants’ participation. InquisitWeb® will host cognitive tasks and will be linked to REDCap using an anonymous identifier. Data from Keenoa® app will be merged with data recording in REDCap using another anonymous identifier. At the end of the study, the principal investigator will retain an electronic copy of the cleaned data set, with all identifying information removed.

### Statistical analysis

**Fig. 2:**
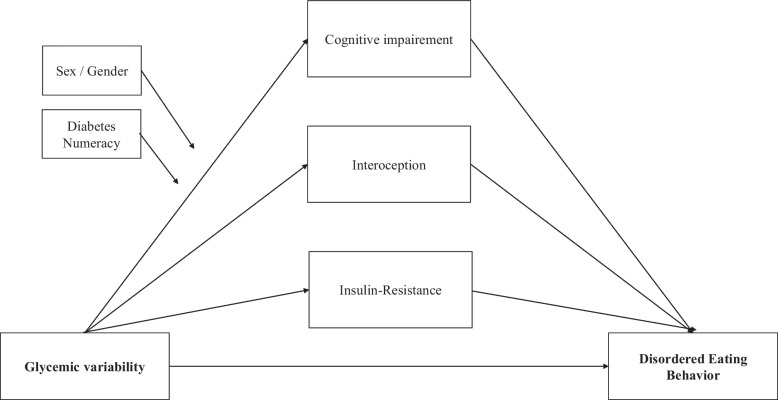
Potential mediation analysis schema with cognitive impairment, interoception and insulin resistance as potential mediators of the relationship between glycemic variability and disordered eating behaviors, using sex/gender and diabetes numeracy as moderators

The sociodemographic characteristics and health outcomes of individuals in the study sample will be summarized with descriptive statistics. Groups will be compared across CV statuses by using the χ2 test for categorical variables and the unpaired *t*-test for continuous variables, or nonparametric equivalent, as appropriate. ANCOVA and generalized linear models will be used to consider potential confounding factors (i.e., sex, BMI, diabetes duration, age of onset, presence of macro- or microvascular complications). Moderation-Mediation analysis will be conducted (PROCESS Macro; Fig. 2). Data curation and preprocessing will be an important part of the statistical plan. To ensure their comparability and interpretability, data will be standardized, or scaling featured, using Scikit-Learn 0.24.2 (a python library). Then artificial intelligence approaches (i.e., LASSO regression or neuronal networks) will be used to explore the potential best predictors of DEB and self-management skills.

### Sample size

The sample size has been estimated based on the cognitive flexibility (one of the most relevant cognitive functions for both disordered eating and self-management skills) effect size computed by Brands et al. [52] in their meta-analysis (*d* = -0.54, SD = 1.07; α = 0.05). Given the potential failure per protocol observed in a similar study design [53], we increased the estimated sample size by 20%. The total number of participants to recruit is established at 150 (75 per group). As an n > 100 is minimally recommended for Least Absolute Shrinkage and Selection Operator, (LASSO) regression, this sample size will also fit for more complex statistical procedures.

### Funding/ethics

This study will be performed in accordance with the Declaration of Helsinki and the study has been approved by the ethics committee of the Centre Hospitalier Universitaire de Québec – Laval University in June 2022 (Reference-No: MP-20–2023-6466). This study has been funded by Diabetes Canada after peer review process (Diabetes Canada End Diabetes:100 Awards Operating Grant; Reference-No: OG-3–21-5571-SI).

## Discussion

This project is groundbreaking in its methodological framework: its use of CGMS and its combination of objective and subjective assessment tools. Moreover, this creative design differs from the current literature in that we start from a biological parameter to identify associated behaviors (whereas most of the studies split the groups into DEB—no DEB). The use of a mixed-method design is also a critical advance. Indeed, the current literature struggles to highlight objective quantitative differences, whereas subjective assessments of PWT1D highlight the importance of cognitive impairment. We believe that we can better understand these discrepancies with this combined approach.

Advances in the web-based infrastructure for research procedures and tools virtualization resulting from experience gained during the COVID pandemic offer us a unique opportunity. Indeed, one of the novel aspects brought by this study is that it can be conducted entirely from home (i.e., online). This is particularly relevant for the second-largest country in the world, which raises the important issue of the inclusiveness of participants living in distant parts of Canada. Therefore, this study will demonstrate the applicability of these new research methods to develop more inclusive studies in the future, especially for territorial disparities.

A better comprehension of the mechanisms and impact of cognitive impairment, especially on DEB and glycemic variability, will result in their earlier detection. This may lead to specific cognitive remediation training, more appropriate communication materials, and thus strengthen patients’ self-management skills. Confirmation of our hypotheses will allow us to study the impact of interventions aimed at reducing glycemic variability (e.g., semi-closed or closed loop system, low carb diet) on brain complications and eating behaviors in PWT1D.

### Study status

Recruitment will begin in September 2022. Based on patient volume at our various recruitment sites, it is estimated that recruitment will take place over 36 months.

## Data Availability

Not applicable.

## Abbreviations

T1D: Type 1 diabetes
PWT1D: Patients with type 1 diabetes
DEB: Disordered eating behaviors
BMI: Body mass index
RDoC: Research domain criteria
CGMS: Continuous glucose monitoring system
CV: Coefficient of variation
eGDR: Estimating Glucose Disposal Rate
SD: Standard deviation
HbA1c: Hemoglobin A1c
